# L1 Cell Adhesion Molecule as a Potential Therapeutic Target in Murine Models of Endometriosis Using a Monoclonal Antibody Approach

**DOI:** 10.1371/journal.pone.0082512

**Published:** 2013-12-04

**Authors:** Cássia G. T. Silveira, Dominique Finas, Peter Hunold, Frank Köster, Katharina Stroschein, Geraldine O. Canny, Gerhard Moldenhauer, Peter Altevogt, Achim Rody, Daniela Hornung

**Affiliations:** 1 Department of Gynecology and Obstetrics, University of Schleswig-Holstein, Campus Lübeck, Lübeck, Germany; 2 Evangelisches Krankenhaus Bielefeld, Department of Gynecology and Obstetrics, Bielefeld, Germany; 3 Clinic for Radiology and Nuclear Medicine, University of Schleswig-Holstein, Campus Lübeck, Lübeck, Germany; 4 Geneva Foundation for Medical Education and Research, Versoix, Switzerland; 5 German Cancer Research Center, Translational Immunology, Heidelberg, Germany; 6 Diakonissenkrankenhaus Karlsruhe Rüppurr, Department of Gynecology and Obstetrics, Karlsruhe, Germany; Institut de Génomique Fonctionnelle de Lyon, France

## Abstract

**Background/Aims:**

The neural cell adhesion molecule L1CAM is a transmembrane glycoprotein abnormally expressed in tumors and previously associated with cell proliferation, adhesion and invasion, as well as neurite outgrowth in endometriosis. Being an attractive target molecule for antibody-based therapy, the present study assessed the ability of the monoclonal anti-L1 antibody (anti-L1 mAb) to impair the development of endometriotic lesions *in* *vivo* and endometriosis-associated nerve fiber growth.

**Methods and Results:**

Endometriosis was experimentally induced in sexually mature B6C3F1 (n=34) and CD-1 nude (n=21) mice by autologous and heterologous transplantation, respectively, of endometrial fragments into the peritoneal cavity. Transplantation was confirmed four weeks post-surgery by *in* *vivo* magnetic resonance imaging and laparotomy, respectively. Mice were then intraperitoneally injected with anti-L1 mAb or an IgG isotype control antibody twice weekly, over a period of four weeks. Upon treatment completion, mice were sacrificed and endometrial implants were excised, measured and fixed. Endometriosis was histologically confirmed and L1CAM was detected by immunohistochemistry. Endometriotic lesion size was significantly reduced in anti-L1-treated B6C3F1 and CD-1 nude mice compared to mice treated with control antibody (P<0.05). Accordingly, a decreased number of PCNA positive epithelial and stromal cells was detected in autologously and heterologously induced endometriotic lesions exposed to anti-L1 mAb treatment. Anti-L1-treated mice also presented a diminished number of intraperitoneal adhesions at implantation sites compared with controls. Furthermore, a double-blind counting of anti-neurofilament L stained nerves revealed significantly reduced nerve density within peritoneal lesions in anti-L1 treated B6C3F1 mice (P=0.0039).

**Conclusions:**

Local anti-L1 mAb treatment suppressed endometriosis growth in B6C3F1 and CD-1 nude mice and exerted a potent anti-neurogenic effect on induced endometriotic lesions *in* *vivo*. The findings of this preliminary study in mice provide a strong basis for further testing in *in* *vivo* models.

## Introduction

Endometriosis is a widely spread multifactorial gynecological disease characterized by the presence of functional endometrial-like tissue in extrauterine locations. It is considered an important women’s health issue affecting about 6-10 % of women of reproductive age and causing a wide spectrum of symptoms mainly related with pain (dysmenorrhea, deep dyspareunia and chronic pelvic pain) and infertility [1]. 

Current treatment strategies for women with endometriosis are symptom oriented and aim at treating chronic pelvic pain and/or infertility. Conservative surgical removal of endometriotic lesions is still the gold standard approach available; however, it commonly provides only temporary pain relief and is associated with high recurrence rates [2]. Being an estrogen-dependent disease, most of the medical therapies aim at inhibiting ovarian activity, resulting in undesirable side effects and rendering their usage less attractive [3]. Therefore, novel therapeutic strategies have been recently investigated mainly targeting the modulation of cellular pathways involved in cell growth, invasion and angiogenesis [4].

In our search for potential molecular markers of endometriosis, we previously identified the L1 cell adhesion molecule (L1CAM, CD171) as a differentially expressed mRNA and protein in endometriotic lesions [5] and proved that it supports endometriotic cell growth, survival, motility and invasiveness, as well as neurite outgrowth [6]. L1CAM is a highly conserved transmembrane glycoprotein of the immunoglobulin superfamily that plays an important role in cell adhesion and motility during the development and regeneration of neuronal tissue [7]. In addition to its physiological role in nervous system development, L1 can also promote other cellular activities by interacting with other CAMs, extracellular matrix molecules, and cell surface receptors, directly and indirectly regulating cell differentiation, proliferation, migration and invasion [8-10].

The connection of L1CAM with various cellular pathways and its cell surface localization renders it an interesting target for a monoclonal antibody-based therapy. Over the past decade, the clinical utility of monoclonal antibodies has been recognized and they are now a mainstay for the treatment of distinct tumors and other human diseases based on their potential anti-proliferative effect [11]. Indeed, the successful application of anti-L1 monoclonal antibody-based therapy in tumors expressing L1CAM has been reported in the literature [12]. Recently, the *in vitro* effects of anti-L1 mAb on endometriotic epithelial cell proliferation, survival, adhesion and invasion have also been shown [6]. 

Given the role of L1CAM as a potential target for anti-cancer therapy and our preliminary data [5,6], we were prompted to investigate the *in vivo* effects of intraperitoneal anti-L1 mAb therapy using two distinct endometriosis mouse models.

## Materials and Methods

### Patients and animal models

Human endometrial tissue samples were obtained from nine women (age distribution: 33.9 ± 7.6) with histologically confirmed endometriosis (rAFS stages I-IV) who underwent gynecological laparoscopy at the Department of Obstetrics and Gynecology, University of Lübeck, Germany. None of the patients had a previous history of endometriosis or were receiving hormone therapy prior to surgery and sampling. All endometrial tissue samples were collected using a Pipelle de Cornier (Laboratoire C.C.D., France) during the mid-proliferative-phase of the menstrual cycle that was estimated using the first day of the last period and posteriorly confirmed by histological analysis. Tissue samples were placed in cold sterile RPMI medium (PAA, Cölbe, GER) containing 100 IU/mL penicillin and 100 IU/mL streptomycin (PAA Laboratories, GE Healthcare Europe, GmbH) and immediately used for *in vivo* studies. Written informed consent was obtained from each patient before surgery, and the study protocol was approved by the ethics committee of the University of Lübeck [Permit Number: 03-068].

Patient characteristics are shown in Table 1.

**Table 1:** Demographic and clinical characteristics of endometriosis patients included in the study.

Patients	Age at surgery (years)	rAFS stage	Reported symptoms
**1^A^**	30	II	Dysmenorrhea, severe pelvic pain, dyspareunia, infertility
**2^C^**	21	I	Dysmenorrhea
**3^C^**	42	III	Dysmenorrhea, severe pelvic pain, dyspareunia
**4^A^**	44	IV	Pelvic pain
**5^B^**	41	IV	Pelvic pain
**6^A^**	28	IV	Dysmenorrhea, dychezia, dysuria
**7^C^**	29	I	Dymenorrhea, severe pelvic pain
**8^B^**	35	III	Dysmenorrhea, severe pelvic pain
**9^B^**	35	I	Dysmenorrhea, severe pelvic pain, dyspareunia, infertility

Endometrial samples that could be ultimately analyzed in the anti-L1 mAb treated (^A^), control (^C^) or both treatment (^B^) groups.

Sexually mature female B6C3F1 (n=34) and CD-1 nude (n=21) mice (aged 4-5 weeks), purchased from Charles River, Sulzfeld, GER, were used for these experiments. The mice were placed in the Laboratory Animal Unit, University of Lübeck, Lübeck, and kept in a pathogen-free and climate-controlled environment with one week of acclimatization prior to experimental proceedings. This study was carried out in strict accordance with the recommendations in the Guide for the Care and Use of Laboratory Animals [NCR (National research council) 1996] and the European Union Council Directive 86/609/EEC. During the experiments, all efforts were made to minimize suffering. The study protocol was approved by the Ministerium für Energiewende, Landwirtschaft, Umwelt und ländliche Räume des Landes Schleswig-Holstein [Permit Number: V312-72241.122-10 (87-8/07)].

### Endometriosis induction and treatment approach

In B6C3F1 mice, endometriosis was surgically induced by transplanting autologous fragments of uterine tissue into the peritoneal cavity. Briefly, each mouse was anesthetized with an intramuscular injection of an anesthetic (1 mg/kg) containing ketamine (100 mg/ml; Ketavet; Pharmacia & Upjohn, Erlangen, GER) and xylazine (2 %; Rompun; Bayer, Leverkusen, GER). Under sterile conditions, a vertical midline incision was made in the lower abdomen. Uterine horns were exposed and carefully resected to preserve the ovaries and endogenous hormone levels. The uterine segment was placed in phosphate-buffered saline at 37 °C, split longitudinally and sectioned into five pieces of 1-2 mm diameter each. These uterine tissue pieces were transplanted into the internal serosal surface of the lateral abdominal wall (right side, n=2; and left side, n=3) and secured with a single non-absorbable suture. They were grafted following a predetermined disposition: two fragments in the left superior abdominal quadrant (below the last left rib and aligned to the spleen), one fragment in the left inferior abdominal quadrant, one piece in the right superior abdominal quadrant, and one fragment in the right inferior abdominal quadrant. The operation was limited to 10-15 min for each mouse to avoid tissue drying out.

The CD-1 nude mice were engrafted with five fragments of eutopic endometrial tissue derived from patients with endometriosis. Fresh human endometrial samples were placed in RPMI medium (PAA, Cölbe, GER), washed twice with Hank’s solution to remove blood and cellular debris and dissected into small tissue fragments of 1 to 2 mm size. The anesthetized nude mice were bilaterally ovarectomized and implanted subcutaneously with a 1.5 mg 60-day estradiol-releasing pellet (Innovative Research of America, USA) in order to abrogate the variation in estrogen cycle and the differences in reproductive endocrinology between the recipient animals and in the transplanted endometrial tissue. Five endometrial tissue pieces were sutured onto the internal serosal surface of the abdominal wall of each ovarectomized CD-1 nude mice following the same orientation described above. Human endometrial samples from each individual donor were transplanted in at least two animals (samples from patients 5 and 8 was transplanted in three and four mice, respectively). Bilateral ovarectomy and subsequent xenotransplantation were completed within 3 h from the time of endometrial tissue collection. 

**Figure 1:**
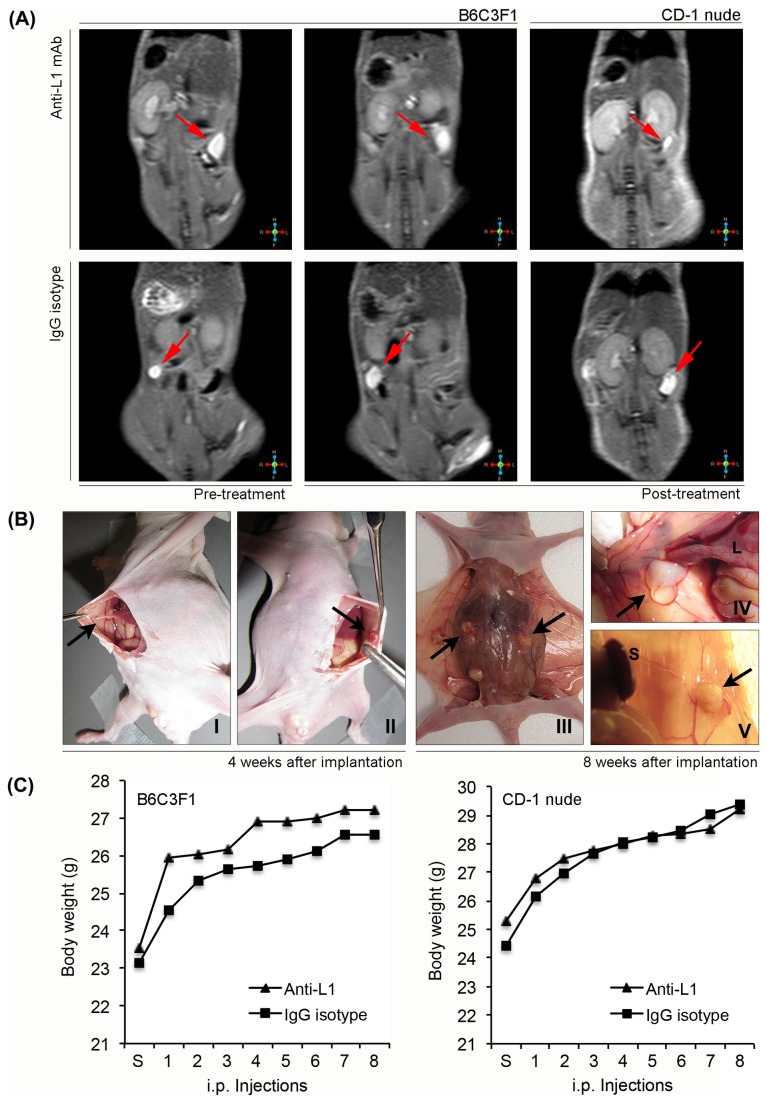
Endometriosis mouse models. (A) *In* *vivo* magnetic resonance imaging (MRI), fat saturated T2-weighted Turbo spin echo images using a dedicated wrist coil at 3T: The red arrows indicate one experimentally induced-endometriotic implant in B6C3F1 and CD-1 nude mice. In a set of B6C3F1 mice, MRI was performed before and after antibody treatment. (B) Laparotomies performed in the CD-1 nude mice 4 (I, II) and 8 (III-V) weeks after the implantation; the black arrows denote the development of two peritoneal endometrial transplants that are shown indicated with arrows in images IV and V after peritoneal layer disruption. (C) Effect of endometrial tissue inoculation and treatment with anti-L1 mAb and control-antibody on body weight.

After the surgical procedures, the animals were individually confined and left for a recovery period of four weeks, allowing ectopic endometrial implants to establish in mice before starting treatment. The presence of experimentally induced endometriotic lesions in B6C3F1 and CD-1 nude mice was subsequently confirmed by *in vivo* magnetic resonance imaging (MRI) (Figure 1A) and by an exploratory laparotomy (second laparotomy) (Figure 1B), respectively. 

B6C3F1 mice were randomly assigned into two treatment groups and treated with rat anti-mouse-L1CAM mAb 555 (n=16; 10 mg/kg; DKFZ, Heidelberg, GER) or rat IgG isotype (n=13; 10 mg/kg; Dianova, Hamburg, GER). The CD-1 nude mice were treated with mouse anti-human-L1CAM mAb 9.3/2a (n=6; 10 mg/kg; a generous gift from Prof. Dr. Peter Altevogt and Dr. Gerhard Moldenhauer, both German Cancer Research Center, Translational Immunology, Heidelberg, GER) or mouse IgG isotype (n=6; 10 mg/kg; Dianova, Hamburg, GER). The nude mice receiving human endometrial tissue from the same donor was equally allocated between the two treatment groups. The respective antibodies and IgG isotype controls were administered twice weekly by intraperitoneal injection over a period of four weeks. The antibody dosage and the frequency of administration were defined based on a former study [13]. Mouse weights were monitored at time of surgery and during the treatment.

### Specimen collection and measurement of lesion volume

Upon treatment completion, mice were sacrificed. Endometrial implants were excised and individually measured in three dimensions (length x width x height in millimeters) using a caliper. The volume of each ectopic endometrial tissue was calculated using the cuboid formula: V (mm^3^) = A x B x C, in which A, B, and C refer to width, length, and height, respectively. The mean lesion volume per treatment group was calculated. All tissue samples were processed (formalin fixed, paraffin embedded (FFPE) and hematoxilin-eosin stained) and histologically evaluated to confirm the presence of endometrial-like structures.

The precision of manual measurements was assessed by comparing the volume calculated with a caliper to the volume data obtained from MRI analyses. For the purpose of this analysis, ten B6C3F1 mice (anti-L1, n=5; placebo, n=5) and twelve nude mice (anti-L1, n=6; placebo, n=6) were imaged immediately prior to sacrifice (Figure 1A).

### Immunohistochemical analysis

The presence of the L1CAM molecule in mouse and human endometrial tissues was assessed by immunohistochemistry using monoclonal antibodies to mouse (clone 555, 1:50) and to human L1 (clone 9.3/2a, 1:50), respectively. The number of proliferating cells and the nerve density within endometriotic lesions were analyzed using polyclonal rabbit anti-proliferating cell nuclear antigen (PCNA) (1:100, Abcam, Cambridge, UK) and monoclonal rabbit-anti neurofilament L (C-term) antibodies (1:100, Epitomics, Inc., USA), respectively. Briefly, serial 4 µm FFPE endometriotic tissue sections on superfrost PLUS slides (Menzel, Braunschweig, GER) were subjected to standard immunohistochemical procedures. After incubation with primary antibodies over night at 4 °C, slides were treated with biotinylated rabbit-anti-rat-IgG (clones 555 and 9.3/2a) or goat-anti-rabbit-IgG (PCNA, neurofilament L) (Vector Laboratories, Inc., Burlingame, USA). Specifically bound antibody was detected using streptavidin-linked alcaline phosphatase (Dianova, Hamburg, GER) and FastRed/Naphtolphosphate (Dako GmbH, Hamburg, GER). Finally, slides were counter-stained with Mayer´s Hematoxilin (Merck KGaA, Darmstadt, GER) and cover-slipped using Faramount Aquaous Mounting Medium (Dako GmbH, Hamburg, GER). For each tissue sample, a serial section was employed as a negative control by omitting the primary antibody. Breast cancer specimens as well as colon and brain tissue sections were used as positive controls. Immunohistochemical reactions were microscopically analyzed at 100- or 200-fold magnification and revised by an experienced pathologist. The immunohistochemistry analyses were performed by two skilled individuals who were blinded to the experimental data.

PCNA positive cells were identified by the presence of nuclear staining. A total of 300 endometrial epithelial cells were counted from representative fields of each lesion at 200-fold magnification. PCNA positive stromal cells were recorded under high power at 10 representative fields of view at 200-fold magnification. Regions containing background or cells with unspecific staining were not considered in the analysis. The percentage of PCNA positive cells was established per each tissue sample. All percentages were used to get the mean value per group.

Anti-neurofilament stained nerves were counted in ectopic implants by analyzing 10 representative fields of view at 200-fold magnification. The average number of stained nerves per field was calculated for each sample and the mean value per group was calculated.

### Statistical analysis

The results were expressed as mean ± SD. Differences between anti-L1 mAb treated and placebo groups were compared using Mann–Whitney U-test. The Wilcoxon rank sum test was used for the paired endometrial samples transplanted in anti-L1 mAb and IgG isotype treated CD-1 mice. P-values < 0.05 were considered significant.

## Results

### Treatment with anti-L1 mAb inhibits the growth of endometriotic lesions

The success and recovery rates after laparotomies in B6C3F1 (autotransplantation) and CD-1 nude (xenotransplantation and posterior exploratory laparotomy) mice were 85 % and 57 %, respectively. In total, five B6C3F1 and nine CD-1 nude mice died before starting treatment because they did not fully recover from the first (B6C3F1, n=5; CD-1, n=5) or second laparotomy (CD-1, n=4). It is possible, that systemic inflammatory challenges, produced by the inflammatory disease endometriosis, compromised the survival of some mice, as this was described before by Dénes et al. [13].

The intraperitoneal administration of anti-L1 mAb and vehicle did not appear to affect the overall health of the mice. No significant changes in weight were observed in either treatment groups (Figure 1C).

**Figure 2:**
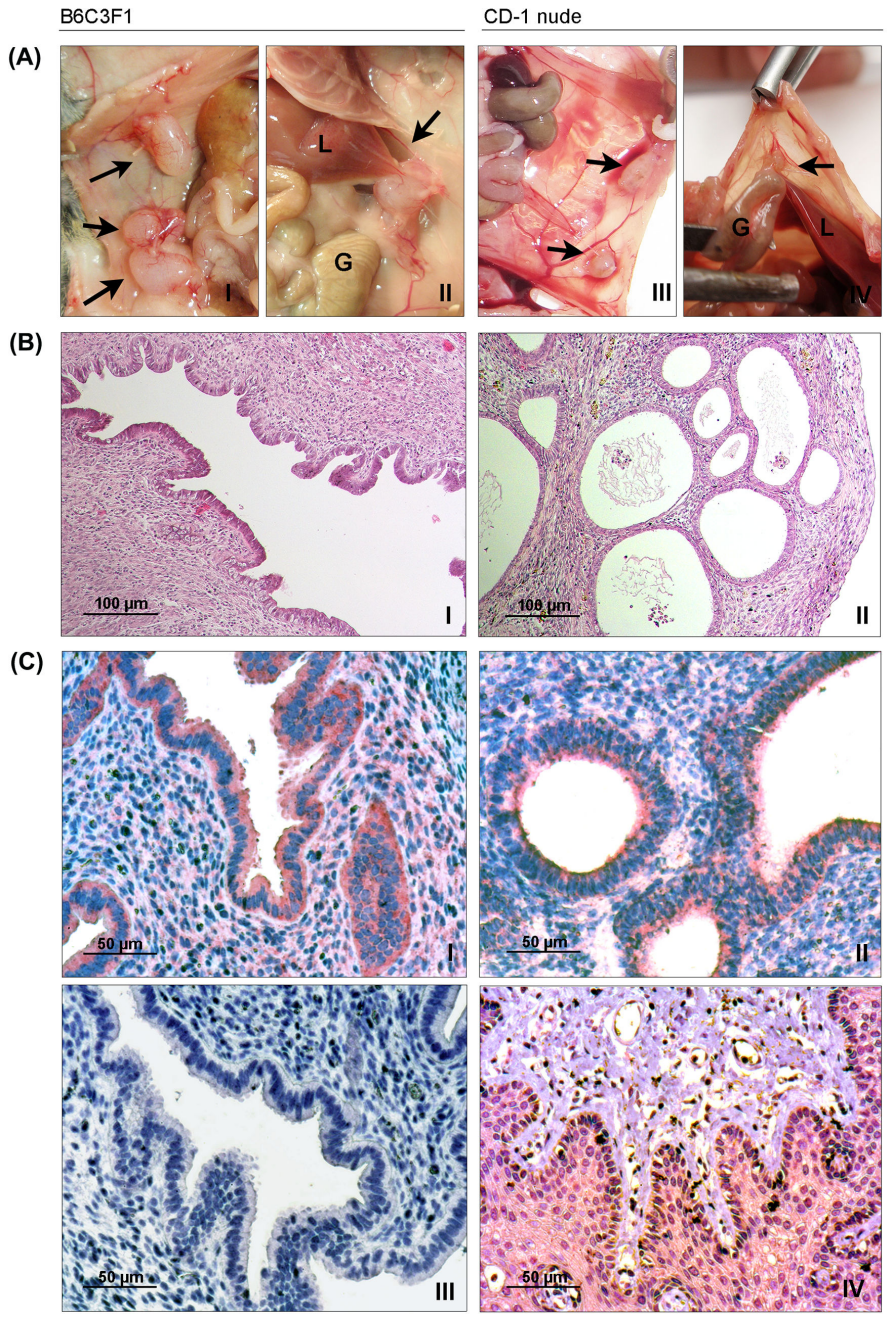
Identification and analysis of induced endometriotic lesions. (A) Four weeks after treatment, B6C3F1 (I, II) and CD-1 nude (III, IV) mice were sacrificed; endometrial implants were identified (black arrows) and excised for further analysis. Adhesions were also observed at implant sites in the B6C3F1 (II) and CD-1 (IV) mice and involved the liver (*L*) and gut (*G*). (B) Hematoxylin-eosin staining of the endometriotic tissues derived from both mouse groups was performed for histological evaluation (magnification: x100). (C) The presence of L1CAM was observed in the endometriotic implants derived from autologous (I) and heterologous (II) models by immunohistochemistry. Negative (III) and positive (colon, squamous epithelium) (IV) controls are shown (magnification: x200).

Upon completion of the treatment, animals were sacrificed and peritoneal endometriotic lesions were excised. Cyst-like structures were detected at the implantation sites, which were co-localized by the presence of a suture (Figure 2A). The average number of recovered lesions detected by MRI and at necropsy was similar in all mouse groups and no significant difference was found between control and anti-L1 mAb treated mice. In anti-L1-treated and control B6C3F1 mice, 86.2 % and 93.8 % of the lesions were retrieved, respectively; in CD-1 nude mice, lesion recovery rate was 100 % in both treatment groups. Histologically, all excised lesions retained both endometrial-type epithelial and stromal cells (Figure 2B) and L1CAM expression (Figure 2C).

Endometriosis-related adhesions in the peritoneal cavity were observed in B6C3F1 and CD-1 nude mice, mainly in those from the control groups (Figure 2A; and Table 2). They were detected at the site of endometriotic implant and mostly involved gut, liver and spleen. Among the CD-1 nude mice, the number of adhesions per mouse was significantly higher in the IgG istotype-treated group compared with the mice that received anti-L1 mAb therapy (P=0.041). In contrast, no significant difference in the number of adhesions between IgG isotype- and anti-L1 mAb-treated B6C3F1 mice was observed.

**Table 2:** Post-surgical endometriosis-related adhesions observed at necropsy.

Mouse strains	Treatment groups	Nr. of mice with adhesions	Nr. of adhesions per group^†^
**B6C3F1**	Anti-L1 (n=16)	6	1.000 ± 0.387
	IgG isotype (n=13)	8	1.077 ± 0.383
**CD-1 nude**	Anti-L1 (n=6)	3	1.000 ± 0.632*
	IgG isotype (n=6)	6	3.167 ± 0.654

^†^Values indicated as mean ± SD; *P<0.05, compared with control group.

**Figure 3:**
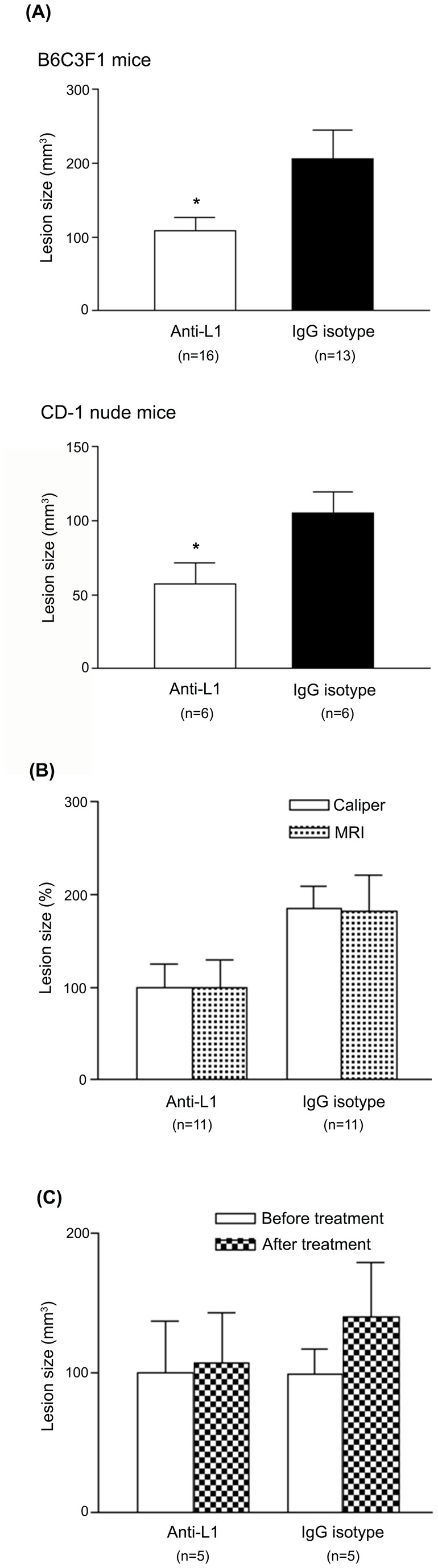
Volume of induced endometriotic lesions. (A) The mean volume of endometriotic implants measured after antibody therapy. Lesion volume was significantly reduced in anti-L1 mAb-treated mice compared with control antibody-treated mice in both B6C3F1 and CD-1 nude groups (*P<0.004). The data shown represent the mean +/- SD. (B) No significant difference between lesion volumes determined by caliper and MRI measurements was observed in anti-L1-treated (100.0 ± 24.97 and 100.0 ± 30.42, respectively) and control antibody-treated mice (184.7 ± 24.83 and 182.6 ± 39.26, respectively), confirming the accuracy of manual measurements. (C) In a set of B6C3F1 mice, MRI measurements of induced-endometriotic lesions in anti-L1-treated and control antibody-treated mice were conducted before (100.0 ± 37.24 and 100.0 ± 17.24, respectively) and after (108.0 ± 35.77 and 140.8 ± 39.12, respectively) treatment.

In all groups, the volume of each endometriotic implant was manually measured. The mean volume of endometriotic lesions was significantly smaller in the anti-L1 treated B6C3F1 mice (109.5 ± 16.87) compared to the control group (205.9 ± 38.86, P=0.037) (Figure 3A). Similarly, the mean lesion size in the anti-L1 treated CD-1 nude mice was significantly smaller (57.19 ± 14.28) compared with control antibody-treated animals (105.6 ± 14.20, P=0.039) after four weeks of treatment (Figure 3A). In addition, the comparison of the ectopic lesion size from anti-L1 mAb and IgG isotype treated CD-1 nude mice transplanted with the same endometrial tissue (three pairs of mice derived from patients 5, 8 and 9) showed a reduction of 30 % to 60 % in the volume of the implants subjected to anti-L1 therapy which was not statistically significant due to small sample size (P=0.250).

**Figure 4:**
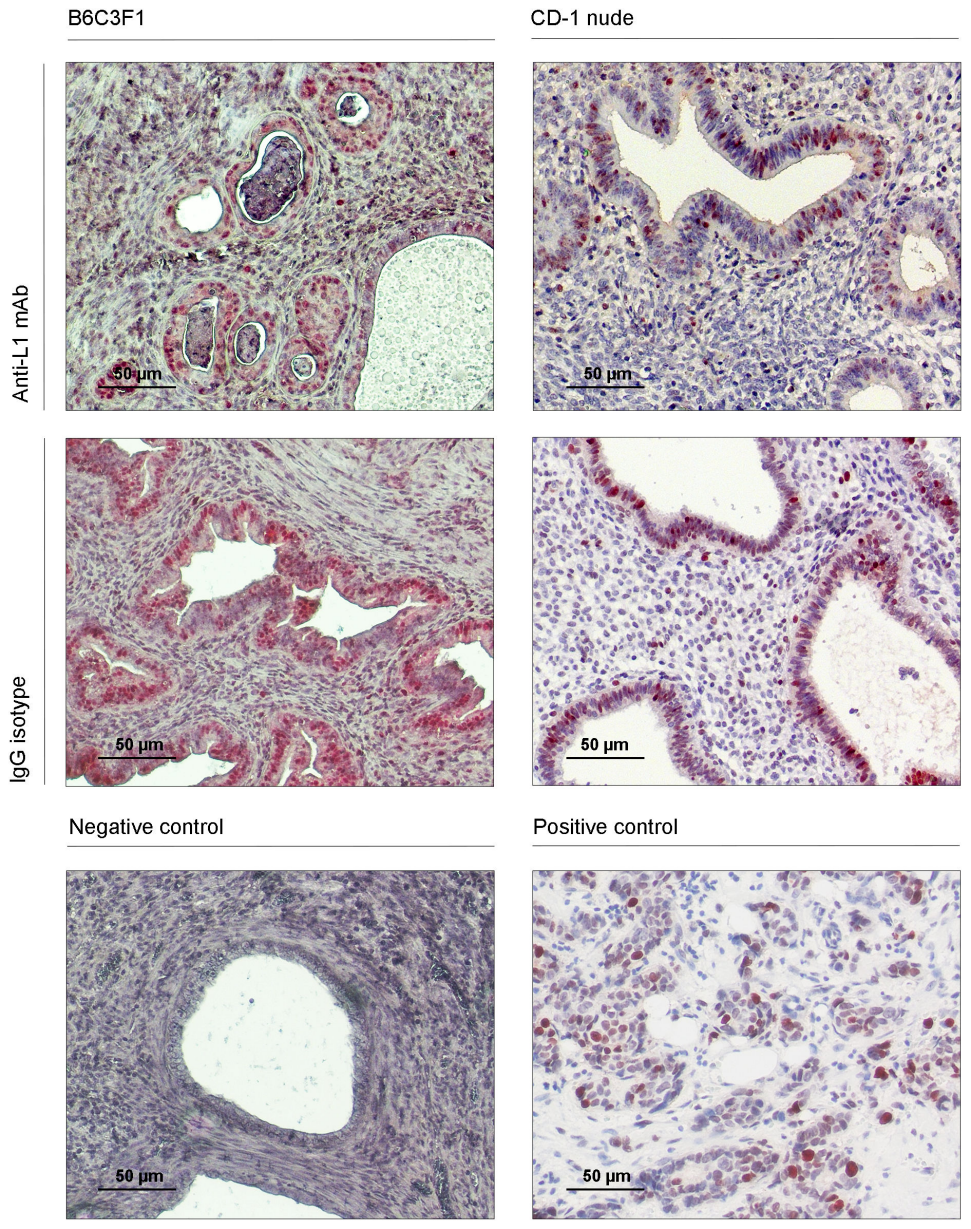
The proliferative status of endometriotic cells in the induced endometriotic lesions assessed by PCNA immunostaining. Negative and positive (breast cancer) controls were included to determine the specificity of the immunostainings (magnification: x100 and x200).

The anti-proliferative effect of anti-L1 mAb therapy was assessed in experimentally induced endometriotic lesions by quantifying the number of proliferating cells immunohistochemically stained with anti-PCNA antibody (a well-established proliferation marker), (Figure 4 and Table 3). In the autologous model, the lesions exposed to anti-L1 mAb treatment had a significantly lower percentage of PCNA positive endometriotic epithelial (13.92 ± 1.809 vs. 22.47 ± 1.865; P=0.002) and stromal (19.11 ± 3.736 vs. 33.10 ± 4.797; P=0.0105) cells compared with control specimens. Similarly, a significantly reduced number of PCNA stained epithelial cells was also detected in the endometrial glands of lesions from anti-L1-treated CD-1 nude animals in comparison to controls (6.98 ± 0.935 vs. 17.34 ± 2.529; P=0.0016), while a trend towards the decrease of proliferating stromal cells was observed in the lesions subjected to the anti-L1 mAb therapy (11.56 ± 2.316 vs. 22.04 ± 4.585; P=0.0789). Paired analysis of the endometrial growths of the same origin also demonstrated a reduction of 10 % to 40% in the proliferative epithelial and stromal cells in the anti-L1 mAb treated lesions compared with the control samples (P=0.0200). 

**Table 3:** Proliferating cells and nerve density in endometriotic lesions in the anti-L1 treated and control groups.

Mouse strains	Treatment groups	Proliferating epithelial cells^†^	Proliferating stromal cells^††^	Nerve density^††^
**B6C3F1**	Anti-L1	13.92 ± 1.809**	19.11 ± 3.736*	3.42 ± 0.246**
	IgG isotype	22.47 ± 1.865	33.10 ± 4.797	4.17 ± 0.273
**CD-1 nude**	Anti-L1	6.98 ± 0.935**	11.56 ± 2.316	3.24 ± 0.429
	IgG isotype	17.34 ± 2.529	22.04 ± 4.585	5.089 ± 0.854

Values indicated as mean ± SD. ^†^Percentage of PCNA positive epithelial cells; ^††^analysis of 10 representative fields of view; *P<0.02, **P≤0.002, compared with control group.

To assess the accuracy of manual measurements, we compared the lesion volume measured using a caliper with the *in vivo* MRI-based measurements obtained before animal sacrifice. No significant difference between these two methods was observed (Fig. 3B). Using this MRI data, we could also assess the lesion size before and after monoclonal antibody treatment in this B6C3F1 mouse subgroup. We observed that anti-L1 mAb treatment did not reduce lesion size but substantially suppressed endometrial implant growth (Figure 3C).

### Anti-L1 therapy reduces nerve density in endometriotic lesions

**Figure 5:**
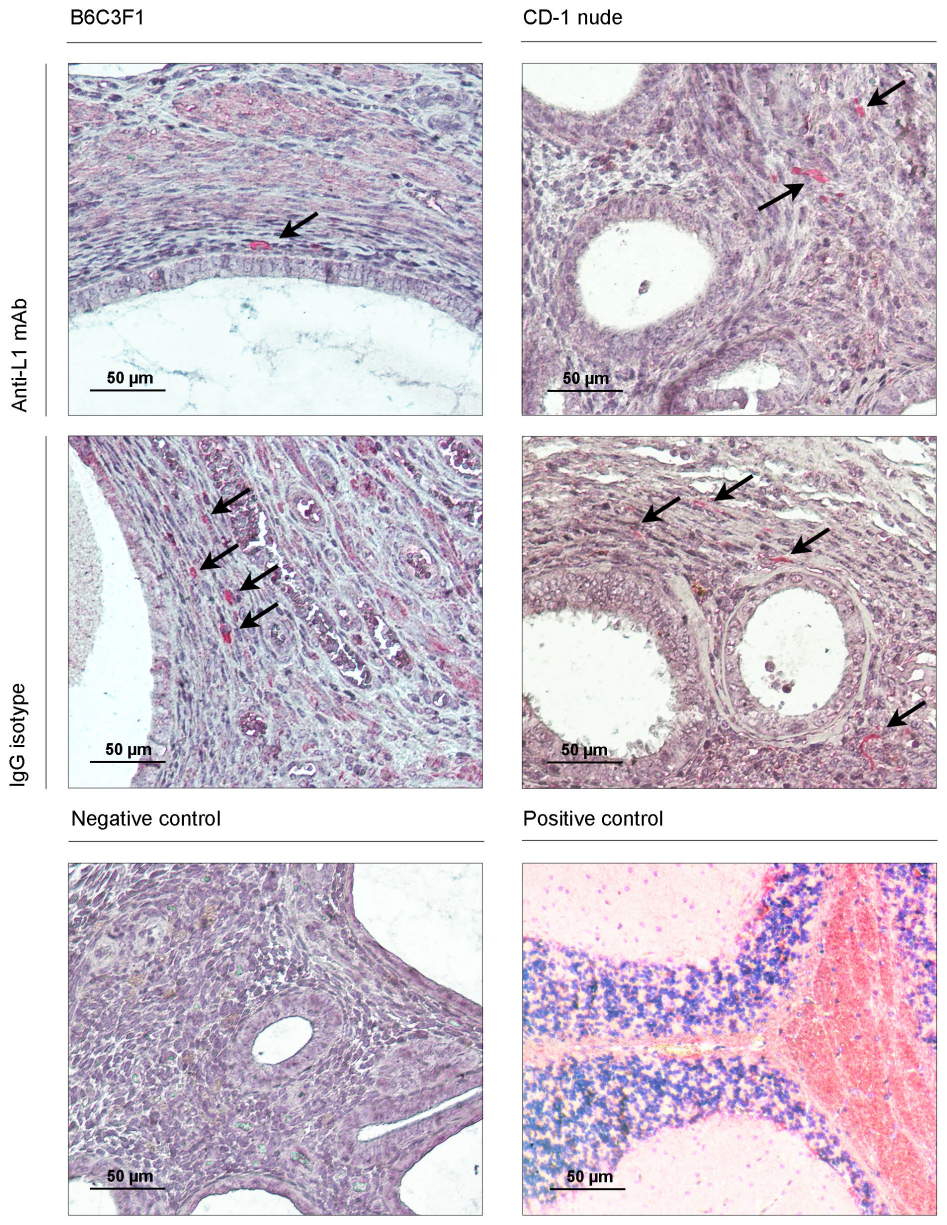
Nerve density in the induced endometrial implants demonstrated by performing immunohistochemistry for neurofilament (NF) L. Black arrows indicate the stained nerve fibers. Negative and positive (brain tissue) controls were included to assess the specificity of the immunostainings (magnification: x100 and x200).

The association between L1CAM expression and neurite outgrowth shown in a previous study [5] led us to test the hypothesis that *in vivo* anti-L1 mAb therapy could also inhibit nerve growth within experimentally induced endometriotic lesions. To assess the potential anti-neurogenic effect of this treatment, we quantified the number of nerves positively stained by monoclonal anti-neurofilament antibody (a well-known neuronal marker) in endometriotic samples collected from each treatment group (Figure 5 and Table 3). In autologous models, our results showed that endometriotic growth exposed to anti-L1 mAb treatment exhibited a significant reduced number of stained nerves per field of view compared to lesions of the control antibody-treated group (3.42 ± 0.246 vs. 4.17 ± 0.273, P=0.0039). A trend towards a diminished nerve density was also noted in endometrial lesions from anti-L1-treated CD-1 animals in comparison to controls (3.24 ± 0.429 vs. 5.089 ± 0.854; P=0.07). Analysis of paired endometrial samples transplanted in CD-1 mice from both treatment groups showed no significant difference in nerve density (P=0.200). 

## Discussion

The present study provides, for the first time, *in vivo* evidence of the feasibility of the L1-directed interference as a therapeutic strategy for the treatment of endometriosis. Intraperitoneal administration of anti-L1 mAb led to a significant inhibition of endometrial implant growth and innervation as well as a reduction of intraperitoneal adhesions in auto- and xenotransplantation mouse models of endometriosis. This apparent disruption of the progression of induced endometriosis supports the anti-proliferative effects of this approach previously observed in an *in vitro* endometriosis model [6]. Likewise, the reduction of nerve density within endometriotic lesions suggests the anti-neurogenic effect of anti-L1 mAb and possibly its ability to reduce endometriosis-related pain.

So far, the importance of the adhesion molecule L1CAM as a therapeutic target has been widely recognized in various tumor entities [14-16] and recently suggested in endometriosis [6]. In fact, several features qualify L1CAM as a novel, attractive target molecule for antibody-based therapy in endometriosis. Apart from its favorable transmembrane location, L1CAM was shown to be expressed in ectopic endometrium, especially in atypical lesions [5], and biologically important in endometriosis pathogenesis driving cell motility, invasion and growth promotion [6]. Moreover, the expression of L1CAM in adults is quite restricted [17,18] and it seems to be especially enhanced in an epithelial-mesenchymal transition-like fashion under particular conditions including inflammation [19,20]. This may possibly explain the detection of L1CAM in human endometriotic cells [5] and in established endometriotic lesions induced in murine models. 

This preliminary study aimed to test the *in vivo* effect of anti-L1 monoclonal therapy in established endometriotic implants which is particularly relevant considering that most patients consult for pain symptoms or subfertility when endometriotic lesions are already well established. Using a similar treatment regime that was formerly shown to be effective for treating ovarian tumors *in vivo* [14], we observed significantly diminished peritoneal implant growth and a decreased number of proliferating cells in endometriotic lesions in the anti-L1 treated mice compared with controls, indicating the ability of anti-L1 mAb to suppress endometriosis progression *in vivo*. This might be especially valuable for those patients whose endometriosis cannot be fully solved by surgery as well as for those individuals with apparent progressive disease characterized by endometriosis relapses. Notwithstanding, as L1CAM overexpression was shown to be particularly related with atypical endometriotic lesions [5], it is possible that anti-L1 mAb treatment might be most effective only for patients with atypical endometriosis. Therefore, the potential use of anti-L1 mAb treatment in preventing endometriosis progression and recurrence deserves further investigation 

The *in vivo* anti-proliferative effect of anti-L1 mAb treatment observed in our study was consistent with previous studies in which the inhibition of L1 expression levels affected tumor growth [12]. Indeed, on a functional level, both soluble and non-soluble L1CAM forms can promote cell proliferation, survival and migration through binding to a variety of heterophilic ligands such as growth factors and integrins, and, consequently, mediate growth factor signaling events as well as enhanced cell adhesion [8,10,21,22] and angiogenesis [23]. Hence, we cannot rule out that the anti-growth effect of anti-L1 mAb observed in this study is based not only on the inhibition of proliferative properties of L1CAM, but also on the reduced adhesive and angiogenic properties of the L1 molecule on endometriotic cells after antibody treatment. So far, L1-related cell adhesion and angiogenesis have been described [23-25] and might be involved in the development of endometriosis and intraperitoneal adhesions, particularly influencing cell growth, attachment and local angiogenesis. Thereby, the suppression of endometriosis growth and adhesion formation observed in our mouse models may possibly be attributed to such functions.

Another important effect of anti-L1 antibody therapy observed herein was related to its ability to prevent neurite outgrowth which has been previously reported [26-28]. Indeed, L1CAM is known to mediate the development and plasticity of the nervous system, including peripheral nerve expansion [29]. In particular, this adhesion molecule can act as both a transducer of nerve-growth–promoting signals and as an effector of neuronal growth by interacting with the cytoskeletal machinery during neuron outgrowth [30-32]. Therefore, the significant decrease in the density of nerves detected in endometriotic lesions of anti-L1 treated mice is possibly due to the inhibition of L1 neurogenic activity and corroborates previous data suggesting such a role [5]. 

In fact, it has been speculated that endometriotic lesions can develop their own nerve supply [33-37] which was demonstrated to be potentially associated with endometriosis-related pain severity [35]. Indeed, persistent nociceptive input from endometriotic lesions is thought to lead to central sensitization [38,39] and possibly to incite neuropathic pain [40]. Hence, it is conceivable that the diminished nerve density in endometriotic lesions observed after local anti-L1 mAb therapy could also contribute to the reduction of endometriosis-related pain symptoms. The effects of this treatment on endometriosis-related pain perception therefore merit further investigation.

The limitation of murine models, especially dissimilarities between mouse and human reproductive physiology must be acknowledged. Despite these limitations, these well-established endometriosis mouse models can offer significant advantages. Besides the limited costs and the opportunity to perform studies in groups of genetically similar animals, long-term studies can be conducted. Thus, mouse models are well suited for the initial investigation of effects of therapeutic drugs and chemicals [41]. Furthermore, it is accepted that surgically induced endometriotic lesions in both auto- and xenotransplanted murine models are similar to the spontaneous disease in women, exhibiting the classic morphological characteristics of endometriosis and analogous gene expression profiles [42,43]. Most interestingly, distinct aspects of human endometriosis can be better simulated by the use of these two murine models, particularly the impact of the immune system on disease development in the autologous models and the molecular and physiological peculiarities of the human eutopic endometrium in immunocompromised mice. 

Second, the safety of anti-L1 mAb should be considered. Although it was not the purpose of this study to test toxicity of this treatment, it is of relevance that no mice died after initiation of antibody therapy, which also did not result in weight loss or other phenotypic alterations compared with controls. Whether anti-L1 mAb may affect physiological angiogenesis and epithelial integrity in the reproductive organs was not tested in these experiments. Moreover, the potential teratogenicity of this anti-neuroangiogenic therapy in other organs and in case of pregnancy also deserves consideration. Nevertheless, it is worth mentioning that the normal distribution of L1CAM in adults is quite restricted [17,18] and might support its suitability as therapeutic target for endometriosis (especially atypical endometriosis) in optimal doses. Thus, an issue to be explored in the future is the lowest effective dose in order to achieve a therapeutically relevant outcome and counteract possible side effects.

Finally, the concept of local anti-L1 mAb therapy for peritoneal endometriosis must be further investigated and possibly compared to other modes of administration. Although intraperitoneal injection is preferred for testing the effects of systemic drugs and fluids in animal models (due to the ease of administration compared with other administration methods), a non-invasive method would be preferable. As the anti-L1 monoclonal antibody is compatible with several pharmaceutical compositions, it can be accordingly formulated and administered via a variety of routes, including oral administration as well as subcutaneous, intramuscular and intravenous injections. 

In summary, this study demonstrates the efficacy of anti-L1 mAb therapy in endometriosis mouse models and offers encouraging evidence that this approach may be valuable in treating endometriosis patients, suppressing the progression of the disease and potentially reducing endometriosis-related pain. The present findings represent only a first step towards potential use of anti-L1 mAb in the treatment of endometriosis. To date, although the safety of anti-L1 mAb remains to be thoroughly tested, its apparent low toxicity may suggest its usefulness as a long-term therapy and may open a new perspective for endometriosis management. The data obtained in this preliminary study provide a rationale for further testing in *in vivo* endometriosis models.
